# Sensitivities to global change drivers may correlate positively or negatively in a foundational marine macroalga

**DOI:** 10.1038/s41598-019-51099-8

**Published:** 2019-10-10

**Authors:** Balsam Al-Janabi, Martin Wahl, Ulf Karsten, Angelika Graiff, Inken Kruse

**Affiliations:** 1 GEOMAR Helmholtz Centre for Ocean Research Kiel, Department of Marine Ecology, Duesternbrooker Weg 20, D-24105 Kiel, Germany; 2 University of Rostock, Institute of Biological Sciences, Applied Ecology and Phycology, Albert-Einstein-Strasse 3, D-18059 Rostock, Germany

**Keywords:** Ecology, Plant sciences, Ocean sciences

## Abstract

Ecological impact of global change is generated by multiple synchronous or asynchronous drivers which interact with each other and with intraspecific variability of sensitivities. In three near-natural experiments, we explored response correlations of full-sibling germling families of the seaweed *Fucus vesiculosus* towards four global change drivers: elevated CO_2_ (ocean acidification, OA), ocean warming (OW), combined OA and warming (OAW), nutrient enrichment and hypoxic upwelling. Among families, performance responses to OA and OW as well as to OAW and nutrient enrichment correlated positively whereas performance responses to OAW and hypoxia anti-correlated. This indicates (i) that families robust to one of the three drivers (OA, OW, nutrients) will also not suffer from the two other shifts, and vice versa and (ii) families benefitting from OAW will more easily succumb to hypoxia. Our results may imply that selection under either OA, OW or eutrophication would enhance performance under the other two drivers but simultaneously render the population more susceptible to hypoxia. We conclude that intraspecific response correlations have a high potential to boost or hinder adaptation to multifactorial global change scenarios.

## Introduction

Marine primary producers contribute about 50% of the world’s carbon fixation^1^. In coastal regions, seaweeds contribute a substantial proportion to this production and to sequestration^2^, but are threatened at a global scale^3^. Many seaweeds are forced to either perish or shift their distributional range^4^ if their acclimation and adaptation to a changing environment is not fast enough. Faced with rapid and multifactorial global change, naturally diverse populations of marine primary producers will respond differently from those with low diversity^5^, as the multifactorial nature of stressors and the intraspecific diversity of populations do interact. Thus, the outcome of simultaneous or sequential selection by multiple drivers will depend on whether the variation in sensitivity among individuals towards drivers A and B are independent of each other or, rather, correlate positively or negatively^6,7^. Positive correlation of sensitivities towards two or more drivers would accelerate positive selection of individuals which are robust to compound change^8^. Conversely, negative correlation would limit adaptive evolution^9^. Negative correlations among sensitivities may further indicate that adaptation in one fitness-trait comes at a cost of another trait by evolutionary trade-offs^10^.

There is an urgent need to experimentally explore how different functionally important traits are impacted by synchronously or sequentially acting multiple drivers in a natural context^11^. This will improve understanding and predicting global change impacts on acclimation and adaptation processes in marine populations^12–14^. Fluctuating drivers may facilitate the selection of robust populations^15,16^. As selection acts on the phenotype, both plastic and adaptive components of the phenotype may contribute to acclimation and adaptation potentials in marine organisms^17^. Response correlations among traits measured on phenotypes are thus only relevant for adaptation potentials if they are based on genetic correlation or functionally related, heritable traits^14,18^.

Many aquatic ecosystems presently face a simultaneous shift of several environmental drivers such as temperature (leading to ocean warming “OW”), pCO_2_ and pH (leading to ocean acidification “OA”), nutrients (leading to eutrophication) and pO_2_ (leading to hypoxia). Interactions among multiple abiotic factors may be complex^19^. Additionally, single factors like OA can have contrasting effects since elevated CO_2_ stimulates photosynthesis but low pH may negatively impact marine algae^20,21^. Also, simultaneous *versus* sequential acting of two factors may produce fundamentally different impacts^6^. At a more regional scale, many coastal ecosystems worldwide are additionally challenged by increasing eutrophication and low oxygen^22^. Interactive effects between OW and regional eutrophication have been reported^23^. Thus, increased organic sedimentation after a fertilization-boosted algal bloom in combination with a more stable stratification (and reduced vertical mixing) due to OW^24^ will lead to larger and more persistent hypoxic zones^25^. Upwelling events sporadically transport this hypoxic, acidified, nutrient rich and cooler waters to shallow habitats^26^. During the course of a year, these drivers may occur sequentially (but not independently) with enhanced nutrient inflow in spring, OW and OA in summer and hypoxic upwelling in autumn (pers. obs.).

The seaweed *Fucus vesiculosus* is a foundational species of the intertidal and shallow subtidal habitat in the North Atlantic including the Baltic Sea. It is highly sensitive to ocean warming and, at the adult stage, features reduced performance (photosynthesis, growth, reproduction, chemcial antigrazing defense) above 16–20 °C and enhanced mortality above 27 °C^27,28^. CO_2_ enrichment, in contrast, rather benefits this species at the adult stage^29^. At the physiological level, linkages between OA and OW with regard to photosynthesis, enzyme systems and carbohydrate production exist in seaweeds^20,30,31^, but compound effects of acidification and OW on marine algae are largely unexplored^32^.

Early life-stages which tend to be more sensitive to global change compared to adults^33^ may, however, represent the bottleneck for the persistence of seaweed species^34^. Studies on the impact of multifactorial pressures on all life stages in seaweeds are still rare^3^. Additionally, while the number of crossed factorial OA and OW experiments has been increasing in the last years^35^, few efforts have been made to consider the role of genetic variation for the adaptive potential of key species (but see^36,37^). Species with short generation times (e.g. phytoplankton) may adapt to new environments fast enough for evolutionary rescue under global change^38^, although initial adaptation responses may not always be projected to long term evolutionary adaptation^39^. For species with generation times of several years, however, examples of fast adaptation are mostly restricted to field observations of selection unrelated to global change factors^40^. In the case of seaweeds with a generation time of at least one year, this lack of knowledge contrasts with their recognized ecological importance as ecosystem-engineers^41^. We recently reported considerable variation among the responses to OA x OW among families of *F. vesiculosus* germlings^42^. This response variation (i.e. genotype-environment interaction sensu^43^) is a prerequisite for directional selection by global change factors, especially so in species such as *F. vesiculosus* with limited dispersal range^14^.

In the present study, we created families of germlings from single parental pairs of *F. vesiculosus*. This set-up allowed us to assume that genetic differences within these families were substantially lower than among families. Among-family differences in performance in common-garden experiments are very likely based on genetic differences. The germling families were exposed to four different global change factors (see below) to explore (1) whether sensitivity differs among families and (2) whether sensitivities towards different drivers correlate positively or negatively or not at all. As a theoretical basis for our experiments we used the conceptual model of Vinebrooke *et al*.^7^, who theoretically explored the importance of co-tolerances towards multiple stressors for the persistence of species and ecosystem functioning. In Vinebrooke’s.^7^ model, positively and negatively correlated sensitivities are postulated to increase, respectively decrease, species persistence under stress relative to a scenario with independent sensitivities. In our study, we ‘replaced’ Vinebrook *et al*.’s.^7^ “species” with our genetically different families and assessed their growth and survival when exposed to OW, OA, nutrient enrichment and/or hypoxia.

Based on previous findings that (i) OA enhanced *F. vesiculosus* germling growth during late summer when combined with OW and (ii) genotypic variation influenced tolerance to both OW and OA^42^ we hypothesized that the sensitivities in *F. vesiculosus* families to OW, OA, nutrient enrichment and/or hypoxia are not independent but rather correlate positively or negatively. We, indeed, found positive correlation among families of their sensitivities towards OA and OW, and towards OAW and nutrient enrichment. In contrast, sensitivities towards OAW and hypoxia correlated negatively. With reasoning analogous to Vinebrook’s^7^, we postulate that correlations in between-family differences in performance responses to global change drivers can have equivalent effects: those families combining high tolerances to multiple drivers have higher likelihood of persistence whereas families with low tolerances are dying-out more likely.

## Results

**Figure 1:**
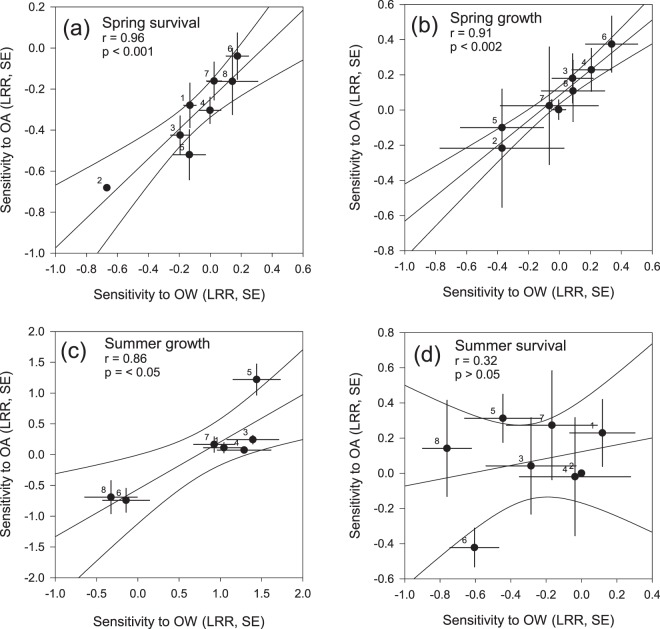
Relationship of family-based sensitivities with respect to warming and acidification (**a**,**b**) growth and survival in spring 2013 (83 days) and (**c**,**d**) growth and survival in summer 2013, (46 days), respectively). For each family, sensitivities towards each treatment factor were calculated in relation to the responses under ambient conditions (log effect ratios “LRR”, means for each family from *n* = 3, standard error “SE”). Mean families’ sensitivity to warming were correlated to their sensitivity to acidification. In spring, the correlation was calculated for 8 families, in summer for 7 families. p-value and correlation coefficient *r* indicated. Dot labels indicate family identity. Modified after^46^.

Sensitivities towards OW and OA showed a significantly positive correlation in spring with regard to the response “survival” (Fig. 1a) and in spring and summer with regard to the response “growth” (Fig. 1b,c) (Pearson’s correlation, survival in spring: r = 0.961, n = 8, p < 0.001; growth in spring: r = 0.911, n = 8, p < 0.002; growth in summer: r = 0.857, n = 7, p < 0.05). In summer, sensitivities of survival towards warming and OA did not correlate significantly (r = 0.318, n = 7, p > 0.05) (Fig. 1d).

**Figure 2:**
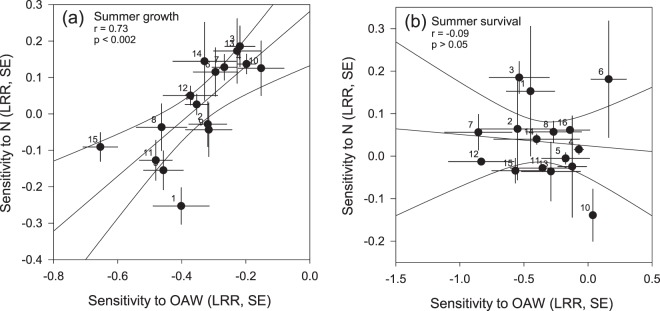
Relationship of family sensitivities towards combined OAW (ocean acidification and warming) and nutrient enrichment (N) in summer 2014 (61 days) for 16 families with regard to growth (**a**) and survival (**b**). For each family, the sensitivities towards each treatment factor was calculated in relation to the performance under ambient conditions (log effect ratios “LRR”, means for each family from *n* = 3, standard error “SE”). p-value and correlation coefficient *r* from Pearson Product Moment correlations are indicated. Dot labels indicate family identity. Modified after^46^.

Sensitivities of growth towards OAW and nutrient enrichment in summer correlated positively (Pearson’s correlation, r = 0.728, p < 0.002, Fig. 2a). There was no correlation of the sensitivities towards OAW and nutrient enrichment, regarding survival (Pearson’s correlation, r = −0.09, p > 0.05, Fig. 2b).

**Figure 3:**
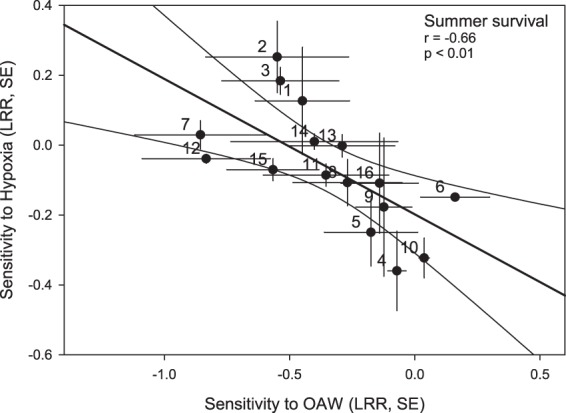
Relationship of family sensitivities towards OAW (ocean acidification and warming) and hypoxia in 16 families with regard to survival. For each family, the sensitivities towards each treatment factor were calculated in relation to the responses under ambient conditions (log effect ratios “LRR”, means for each family from *n* = 3, standard error “SE”). p-value and correlation coefficient *r* from Pearson Product Moment correlations are indicated. Dot labels indicate family identity. Modified after^46^.

Sensitivities of survival towards OAW and hypoxia correlated negatively in late summer (Pearson’s correlation, *r* = −0.66, p < 0.01, Fig. 3). Growth response was not analysed because this experiment only lasted for 3 days.

## Discussion

The present study is the first to show for a marine primary producer the existence of positive correlations between performance responses towards ocean acidification (OA) and ocean warming (OW), and between combined ocean acidification and warming (OAW) and nutrient enrichment. Furthermore, negative correlations were found between the former sensitivities (specifically towards OAW) and the families’ sensitivity towards hypoxia. Since these correlations are identified among genetically different families of germlings in common garden experiments, it is reasonable to assume that these correlated phenotypic responses have a genetic component. From this, two alternative interpretations arise. First, those phenotypes which grow and survive well under OA, OW, OAW and nutrient enrichment could be just of good ‘individual quality’^44^. Since ranked phenotype performances in diverse populations usually show a linear relationship under environmental parameters, this ‘individual quality’ could be the underlying mechanism responsible for our correlations. A terrestrial study subjecting families of jack pine seedlings to an elevated CO_2_-temperature-nitrogen environment^45^ suggests such ‘individual quality’ dependency by finding (i) significant genotypic differences for height and biomass, (ii) families keeping their rank relative to other families (from the ambient to the elevated CO_2_-temperature) and (iii) significant rank correlations between height of families grown in elevated CO_2_-temperature and height of families at 10 years of age in the field. A study on thermotolerance of two macroalgal species showing that those genotypes that performed well in elevated temperature also performed well in control temperature, additionally support the ‘individual quality’ hypothesis^14^. In our study however, the simultaneous existence of a *negative* correlation between the sensitivities with regard to OAW and to hypoxia, respectively, seem to weaken the ‘individual quality’ hypothesis because the most OAW-resistant phenotypes at the same time are the most hypoxia-sensitive ones. Another explanation for the correlated phenotypic responses is a functional relation or genetic correlation underlying the observed response patterns. In this case, these traits of high performance under OA, OW, OAW and nutrient enrichment may not evolve independently and simultaneous robustness under these combined conditions could be heritable. Then, these positive correlations could represent a ‘fast-forward’ mode for evolution under global change and would clearly increase the adaptive potential of the habitat-forming seaweed *Fucus vesiculosus*.

Correlations between sensitivities towards environmental factors with a verified genetic background are often considered to be based on genetic pleiotropy (when one gene influences two or more unrelated phenotypic traits), or linkage disequilibrium (the non-random association of alleles of different genes at different loci)^8,46,47^. However, for the correlation we found between OW and OA we are not aware of any genetic mechanism which could lead to the described patterns. This is because first, we could not follow a quantitative trait locus (QTL) approach with appropriate breeding design to explore heredity and second, our response variables growth and survival typically integrated many traits and genes, and hence we do not know which and how many traits contributing to our sum variables are influenced by OW and OA^46^. Thus, we can only speculate about the causes for this correlation of responses. One possibility is a functional connection of the two factors in photosynthetic processes, such as any temperature-driven enzyme kinetics towards better utilization of CO_2_ (e.g. RuBisCO, carboanhydrase) which exhibits a higher expression in some families but not in others^46^. Another physiological functional relation for good combined performance under OA and OW could be that some phenotypes can benefit better from CO_2_ fertilisation, enabling them to allocate more energy to heat tolerance than others. A functional linkage between the responses towards combined OAW and nutrient enrichment may be related to a higher or more efficient carbon uptake and fixation under OAW conditions resulting in stronger growth, which, of course, would require more nutrients. In this context, it would be worthwhile to test if functional relations or genetic correlations exist in other marine algae. Diatoms, as close phylogenetic relatives of *Fucus* and most important primary producers in the world oceans could be key candidates to do so. Compared to seaweeds, diatoms have the additional advantage of short generation times facilitating a multi-generation evaluation of the postulated fast evolution. But also the existence of genomes of different diatoms might provide feasible tools to explore the underlying molecular mechanisms.

In contrast to the foregoing, the negative correlation between responses to OAW and to hypoxia has the potential to block adaptation to global change in regions where these drivers occur simultaneously or sequentially. Indeed, those families which survived a summerly OAW stress succumbed more readily to a hypoxic autumn upwelling. A functional link between OAW and hypoxia may exist insofar as genotypes capable of stronger growth and higher survival under OAW may have a higher metabolic rate which goes along with enhanced respiration rates. This presumed higher oxygen demand might lead to higher susceptibility of the germlings to hypoxic conditions when the metabolic balance (i.e. gross primary production relative to respiration^48^) becomes heterotrophic which is the case at night and throughout the day at temperatures exceeding 26 °C^49^. Variation among families in response to OAW and hypoxia may then be imposed by among-genotype variation in metabolic rate and oxygen demand. The responses of the holobiont *F. vesulosus* may also be affected by changes in its associated surface microbiome, as its composition (and possibly function) shifts quite substantially under OW, OA and OAW^50^. All these possibilities are highly speculative however and mainly highlight the need for more indepth research to mechanistically explain the described sensitivity correlations.

**Figure 4:**
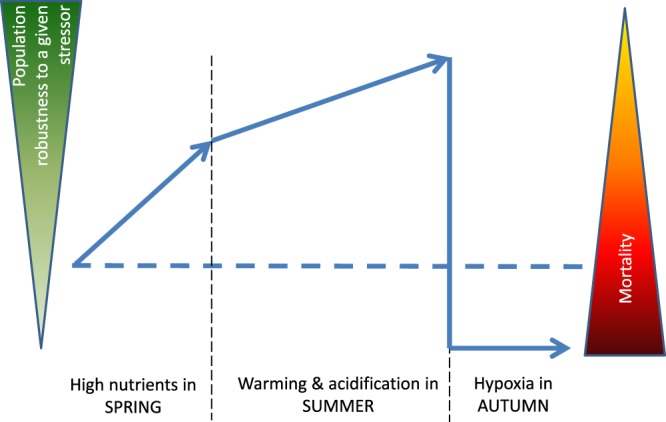
Concept of adaptational boost or bust driven by sequential and/or synchronous stressors. Expected shifts in robustness and mortality under eutrophic conditions (spring), followed by high temperature stress and acidification in summer, followed by hypoxic upwelling in autumn. Sensitivities to the first three factors correlated positively and boost adaptation to nutrient-rich, warm and acidified conditions but at the same time render the population more sensitive to hypoxia. The dashed horizontal line represents the robustness of the non-selected (“naive”) population in the absence of the respective drivers.

The sequential occurrence of stressors in *F. vesiculosus* habitats in the Baltic Sea with OA during spring, OAW during summer and hypoxic upwelling in autumn is common^26,51^. These seasonal fluctuations, possibly interacting with the observed sensitivity correlations, indicate that, in addition to the strength of drivers, their timing plays an important role not only from a physiological^6^ but also from an evolutionary point of view. As proposed in the model of Vinebrook^7^, in a system featuring multiple stress in a dynamic mode like the Western Baltic Sea, ecological history (or stress history *sensu*^52^) matters for the effects of stress. Positive correlations among the sensitivities to OA, OW and nutrient enrichment lead to stress-induced tolerance (of the surviving part of the population) and accelerated adaptation to this set of global change factors. In contrast, negative sensitivity correlations among OAW and hypoxia lead to stress-induced sensitivity and should slow down adaptation to OAW in regions with hypoxic upwelling. In consequence, the described and predicted spread of hypoxic zones in the Baltic (and other coastal regions)^53^ may not only impact seaweeds directly^54^, but additionally lower their capacity to adapt to the ongoing environmental change (Fig. 4). A seasonal sequence of stressors with correlating sensitivities in the target species has a huge potential to accelerate adaptation when the correlations are positive or, in contrast, block adaptation and lead to genetic erosion with all its functional and evolutionary consequences^55^ when correlations are negative. The latter situation resembles the adaptation block by negative correlations among fitness traits^18^.

Our results corroborate the need for investigating responses to multiple stressors for revealing correlations of responses that would be hidden when stressors are analysed separately^12,46^. Most studies also disregard natural variabilities: these are (i) fluctuations in the treatment factors during the experiments, which can have profound effects on experimental results^56^ and (ii) fluctuations in the treatment factors in the natural habitat, since these have determined the evolutionary history and will determine the fate of the populations tested^16^, (iii) temporal variability in intensity of various drivers determining their synchronous *versus* sequential action and (iv) sufficient standing genetic variation between experimental groups or organisms tested. Understanding phenotypic correlation of responses towards multiple environmental factors in the context of natural variability will help to better predict global change impact on marine populations^46^.

## Material and Methods

(Parts of this section were extracted from the doctoral thesis of BAJ^46^).

### Experimental design and course of the experiments

For all experiments, germlings of *Fucus vesiculosus* from full-sibling families were produced following a controlled protocol and reared on sandstones as hard substratum, so that all germlings on one labelled sandstone had always the same mother and father. Each parent contributed to only one offspring-group. This allows us to assume that genetic differences within families were substantially lower than among families (for an equivalent approach see^57^), thereby generating a set of intraspecific between-family diversity, which could be replicated among mesocosms. Sandstones with germlings of one family each were then distributed among the different treatment combinations. Each family was placed into each of the nine mesocosms (described as Kiel Outdoor Benthocosms below), with *n* = 3 replication of the same treatment and family (see experimental design depicted in Fig. S1). This ‘common garden’-setting reduces environmental “noise” and allows assuming that between-family differences in responses to a given treatment had a genetic component^57^.

In spring and summer 2013, eight and seven full-sibling families, respectively, were exposed to a crossed warming (OW) and acidification (OA) treatment, whereas in summer 2014, 16 families were exposed to a combined treatment of warming and acidification (details in^58^), (OAW), crossed with a nutrient enrichment treatment (N) (details in^59^). The 16 families from the ‘all ambient’ treatment regarding temperature, CO_2_ and nutrient levels were subsequently exposed to a hypoxic upwelling experiment.

### Collection and gamete acquisition

To create eight different families of germlings for the experiments in spring and summer 2013, a total of 53 fertile *F. vesiculosus* were sampled in the south western Baltic Sea (Bülk, Germany, 54°27.327′N, 10°11.977′E) at the end of November 2012. To ensure for genetic variation among prospective parents, the distance between individuals sampled was at least 2 meters, which is the estimated maximum dispersal distance of *F. vesiculosus* gametes at calm conditions^60^. Collected algae were transported to the laboratory in cooling boxes. From each alga, all fertile receptacles were cut and their gender was determined under the microscope at 100x magnification (Olympus BH-2). Parental pairs were formed by combining all the receptacles of one male and one female adult *Fucus* individual in a plastic dish. Receptacles were rinsed in tap water, blotted dry and placed in the darkness at 8–10 °C. After 5 days, the receptacles were immersed in 3 L of sand-filtered seawater (salinity of 15–16) and exposed to a photon flux rate of 110 µmol photons m^−2^ s^−1^ (aquarium lamp Osram HQL) for 3 h to allow for gamete release and for egg fertilisation. A homogeneous zygote suspension was created by vigorous stirring, of which 0.67 ml solution was pipetted onto the upper surface of a sandstone cube with 2 cm edge length covered with seawater. After settlement, the density of the germling was on average 126 (SE 53) per cm² of sandstone. Although, settlement was patchy, we chose not to standardize it by weeding because (1) we wanted to keep disturbance at a minimum, (2) sensitivity was expressed relative to the start density on single cubes and (3) start density did not affect survival (Spearman Rank correlation r = 0.21) at this early ontogenetic stage. The sandstone substrata had been immersed in unfiltered seawater for several days to allow the establishment of a microbial biofilm. *Fucus vesiculosus* germlings were cultured in sand-filtered seawater at 8 °C during 8 weeks in a room with windows providing natural dim light conditions (200–400 µmol photon m^−2^ sec^−1^). The dishes with germlings were positioned in a way to ensure similar light conditions. Once a week, seawater was exchanged. After this pre-incubation which served to protect them from grazing during their most vulnerable phase, germlings were transferred to the Kiel Outdoor Benthocosms (KOB) when they were about 0.5 mm long. The KOB are a floating mesocosm system comprised of 12 tanks (1400 L volume) with a continuous flow-through of natural fjord water. Thus, all environmental fluctuations are allowed in the tanks. Treatments (OW, OA, etc) are applied as delta-treatments, i.e. as computer-controlled modifications (warming, acidification…) of the actual *in situ* values (details in^58^).

For the experiments in summer 2014, 16 families were established following the same procedure regarding collection and gamete acquisition but with the following exceptions: here, a total of 64 fertile *F. vesiculosus* (46 females, 18 males) were collected in mid-June 2014, receptacles were stored in the dark for 6 days at 14 °C, 1 ml of a homogeneous suspension of fertilised eggs was pipetted onto the sandstone surface for each family, and all germlings were cultured during 3 weeks at 15 °C until introduction to the KOBs.

### OW, OA, nutrient enrichment and local upwelling treatments

All experiments were performed at a near-natural scenario in the Kiel Outdoor Benthocosms^58^. In spring and summer 2013, *F. vesiculosus* germlings were exposed to all combinations of the two treatment factors temperature and CO_2_ at the two levels “ambient” and “future”. The “ambient” conditions corresponded to the actual *in situ* conditions of Kiel Fjord at 1 m depth transferred to the experimental tanks of the KOB in real time by a continuous flow-through (1 tank-volume per day, i.e. 1500 L 24 h^−1^). ‘Future’ conditions according to the predictions of the year 2110 in the Baltic Sea^61^ were achieved by dynamically adding 5 °C to the actual ambient temperature of the Kiel Fjord and by increasing the CO_2_ concentration in the hooded air headspace of the tanks to 1100 µatm CO_2_. The continuous flow-through assured that the natural fluctuations (Fig. S2) of all environmental variables were mirrored in the experimental tanks in all treatment combinations. Three experimental units of the KOB were run at ambient Kiel Fjord conditions, three units were warmed and three units were acidified, both relative to the ambient fjord conditions. During the *in situ* reproduction periods, sandstones were protected from drifting *Fucus* zygotes by placing them into PVC boxes (70 cm × 40 cm × 12 cm) suspended within the tanks. This set-up ensured the maintenance of the treatments OW and OA by temperature exchange with the main KOB water body through the thin PVC walls (6 mm) and for CO_2_ exchange across their water surface with the atmosphere of the overlying KOB headspace, respectively. Water with the temperature and CO_2_ conditions of the KOB tanks was partially replaced biweekly into the PVC box after filtration through 50 µm mesh to prevent the accidental introduction of new *F. vesiculosus* eggs (about 100 µm in diameter). In mid-June, when the tanks of KOB were serviced, *F. vesiculosus* germlings were stored in indoor mesocosms during 14 days while maintaining the respective temperature and CO_2_ conditions. Temperatures were controlled by using internal heater elements (600 W, Schego Titan, Schemel & Goetz, Offenbach am Main, Germany) and values were set according to the delta treatment. The OA conditions were achieved by bubbling CO_2_ enriched air (1000 µatm CO_2_) directly into the water of the mesocosms. Temperature and pH were logged continuously and, additionally, hand-measured daily; total alcalinity (TA) was assessed every third day and DIC monthly. Measurements of pH, TA and DIC followed the Best Practices for Ocean Acidification Research (Riebesell *et al*. 2011) (details in^62^).

*Fucus vesiculosus* germlings were transferred to their containers in the KOB tanks and allowed to acclimatize for 8 weeks before starting the measurements of the experiment. Growth and survival were measured in spring for the period 02.04.2013–30.05.2013. In summer growth was measured for the period 26.06.2013–08.08.2013 and survival for the period 30.05.2013–01.08.2013.

In summer 2014 (09.07.2014–08.09.2014), the factor nutrient (N) was applied in three tanks along with the combined conditions of ocean acidification and warming (OAW) in another three tanks. Nutrient enrichment, i.e. the increase of the concentrations of NO_2_, NO_3_, and PO_4_, was realized by doubling the mean concentration of each nutrient measured during the last seven years (2006–2013) (Suppl. Table 1). We kept the natural P:N ratio of the Kiel Fjord, which is rather N-limited and off the Redfield ratio. NaNO_2_ (Merck, Germany), NaNO_3_ (Carl Roth, Germany) and H_2_NaO_4_P.H_2_O (ACROS organics, Germany) were dissolved in fjord water 10 minutes before adding them with syringes to the PVC boxes.

In mid-September, 2 months after the OAW & N experiment, the 16 families from the tanks with ambient conditions were subjected to a simulated upwelling event during three days. This was achieved by a continuous flow-through of water pumped from 15 m water depth from Kiel Fjord, which was hypoxic during that period (O_2_ = 2.71 ± 0.37 mg L^−1^, T = 16.35 ± 0.29 °C, pH = 7.403, Sal = 22.8). Acidified conditions, occurring also during upwelling events, were not observed to decrease *F. vesiculosus* survival in previous experiments^54^. Compared to the previous conditions in the KOB, temperature and salinity were not strongly affected by the upwelling treatment (Fig. S3). Hypoxia with a mean (±SD) of 2.75 ± 0.41 mg O_2_ L^−1^ differed from the previous oxygen concentrations under ambient conditions −8.913 ± 0.38 mg O_2_ L^−1^ (Fig. S4) and was considered the most likely driver of the observed effects of the upwelling.

Temperature and pH (to extrapolate CO_2_) were measured daily with a calibrated sensor (pH, Mettler Toledo GmbH, Giessen, Germany) and salinity was measured with a conductivity meter (WTW Cond 3110 + Tetra Con 325, Wissenschaftlich Technische Werkstätten, Weilheim, Germany) (Fig. S2). During the hypoxia treatment, oxygen and temperature were logged every 10 minutes with a Multi WTW Oxy 3515 (Fig. S4), while pH and salinity from 15 m depth were measured on the day before the experiment as described above.

### Survival

Survival of *F. vesiculosus* germlings was determined as the % of surviving germlings between the start (t_0_) and the end (t) of respective experiments: OW & OA experiments in spring and summer 2013; OAW & N experiment 2014; and upwelling experiment 2014. The number of germlings for each experimental population was determined under the binocular at 25× magnification. Germlings of the upper surface of the sandstone (2 cm^2^) were counted. The percentage survival was determined as:

$${\rm{Survival}}\, \% =\frac{{\rm{Number}}\,{\rm{t}}}{{\rm{Number}}\,{{\rm{t}}}_{0}}.100$$

### Growth

Germlings’ mean growth was determined by recording digital images of 10–15 germlings per experimental unit at 40× magnification (SteREO Discovery V8 – Carl Zeiss Jena GmbH) according to the method used by Steen and Scrosati^63^. The side-view of the single germlings was measured by means of image analysis using Image J 1.45 s (National Institutes of Health, USA). For each experimental unit, the mean area of the germlings assessed was calculated. The germling areas were measured at the beginning (Area t_0_) and at the end (Area t) of the experiments (OW & OA experiments in spring and summer 2013; OAW & N experiment summer 2014). Growth was not determined for the hypoxia experiment considering the short duration of three days. Individuals from each experimental population were chosen randomly for measurements since germlings were too small to be labelled. Growth was calculated as the ratio between germling *Area t*_0_ at day 0 and *Area t* after *x* days at the end of the experiment:

$${\rm{Growth}}=\frac{{\rm{Area}}\,{\rm{t}}}{{\rm{Area}}\,{{\rm{t}}}_{0}}\ast x\,day{s}^{-1}$$

### Correlation of sensitivities

Following the conceptual model of Vinebrooke *et al*.^7^, the correlation of sensitivities (assessed as Log Effect Ratios, see below) was explored between factors or factor combinations using Pearson Product Moment Correlations based on the mean response of the three replicates per treatment. When the correlation was positive and significant we interpret this as a positive correlation of the phenotypes in their responses towards these two factors. When the correlation was negative and significant we interpret this as a negative diversity-driven correlation of the phenotypes in their responses towards these two factors. Responses to the single treatments by each family were calculated as described below.

### Calculations of the sensitivities to OW and OA with regard to survival and growth

In all cases, the response to a given treatment was quantified as the Log Effect Ratio (LRR) of the mean performances under the treatment and under ambient conditions. The variability of these responses was expressed as the SE of all individual responses (i.e. the LRR of the performance of a given replicate in a given treatment relative to all the other replicates in the reference treatment)

$${\rm{OW}}\,{\rm{sensitivity}}=\,\mathrm{Log}\,\frac{{\rm{Survival}}\,{\rm{T}}+}{{\rm{Survival}}\,{\rm{T}}-}\,{\rm{at}}\,{\rm{non}}-{\rm{acidified}}\,{\rm{conditions}}\,{\rm{and}}$$

$${\rm{OA}}\,{\rm{sensitivity}}=\,\mathrm{Log}\,\frac{{\rm{Survival}}\,{{\rm{CO}}}_{2}+}{{\rm{Survival}}\,{{\rm{CO}}}_{2}-}\,{\rm{at}}\,{\rm{ambient}}\,{\rm{temperatures}}.$$

$${\rm{OW}}\,{\rm{sensitivity}}=\,\mathrm{Log}\,\frac{{\rm{Growth}}\,{\rm{T}}+}{{\rm{Growth}}\,{\rm{T}}-}\,{\rm{at}}\,{\rm{non}}-{\rm{acidified}}\,{\rm{conditions}}\,{\rm{and}}$$

$${\rm{OA}}\,{\rm{sensitivity}}=\,\mathrm{Log}\,\frac{{\rm{Growth}}\,{{\rm{CO}}}_{2}+}{{\rm{Growth}}\,{{\rm{CO}}}_{2}-}\,{\rm{at}}\,{\rm{ambient}}\,{\rm{temperatures}}.$$

Eight families were used in spring and seven families in summer due to die-off of one family.

### Calculations of the sensitivities to OAW and nutrient enrichment with regard to survival and growth

 

$${\rm{OAW}}\,{\rm{sensitivity}}=\,\mathrm{Log}\,\frac{{\rm{Survival}}\,{\rm{OAW}}+}{{\rm{Survival}}\,{\rm{OAW}}-}\,{\rm{at}}\,{\rm{ambient}}\,{\rm{nutrient}}\,{\rm{conditions}}\,{\rm{and}}$$

$${\rm{Nutrient}}\,{\rm{enrichment}}\,{\rm{sensitivity}}=\,\mathrm{Log}\,\frac{{\rm{Survival}}\,{\rm{N}}+}{{\rm{Survival}}\,{\rm{N}}-}\,{\rm{at}}\,{\rm{ambient}}\,{\rm{temperatures}}\,{\rm{and}}\,{{\rm{CO}}}_{2}.$$

$${\rm{OAW}}\,{\rm{sensitivity}}=\,\mathrm{Log}\,\frac{{\rm{Growth}}\,{\rm{OAW}}+}{{\rm{Growth}}\,{\rm{OAW}}-}\,{\rm{at}}\,{\rm{ambient}}\,{\rm{nutrient}}\,{\rm{conditions}}\,{\rm{and}}$$

$${\rm{Nutrient}}\,{\rm{enrichment}}\,{\rm{sensitivity}}=\,\mathrm{Log}\,\frac{{\rm{Growth}}\,{\rm{N}}+}{{\rm{Growth}}\,{\rm{N}}-}\,{\rm{at}}\,{\rm{ambient}}\,{\rm{temperatures}}\,{\rm{and}}\,{{\rm{CO}}}_{2}.$$

Sensitivities of 16 families were calculated.

### Calculations of the sensitivities to OAW and Hypoxia

 

$${\rm{OAW}}\,{\rm{sensitivity}}=\,\mathrm{Log}\,\frac{{\rm{Survival}}\,{\rm{OAW}}+}{{\rm{Survival}}\,{\rm{OAW}}-}$$

Only those germlings were exposed to hypoxia sensitivity which had experienced ambient conditions previously:

$${\rm{Hypoxia}}\,{\rm{sensitivity}}=\,\mathrm{Log}\,\frac{{\rm{Survival}}\,{\rm{Hypoxia}}}{{\rm{Survival}}\,{\rm{OAW}}-}$$

Sensitivities of 16 families were calculated.

The relationship between the responses to two different drivers were described by the Pearson Product Moment procedure using the mean LRRs of each family with regard to the two drivers.

### Abiotic factors

During the OW & OA experiment in spring and summer 2013, mean temperature (±SD) was 14.3 ± 5.2 °C under ambient conditions, and 18.9 ± 5.7 °C under warmed conditions. The mean difference between the ambient and the warm treatment was 4.5 ± 0.9 °C. The mean pH (±SD) was 8.30 ± 0.32 under ambient conditions and 8.09 ± 0.33 under acidified conditions, with a mean difference of 0.22 ± 0.08. Mean salinity (±SD) under ambient conditions was 14.5 ± 1.3 (Fig. S2). The SDs of these variables mainly reflect the biogenic circadian and the abiotic seasonal and stochastic variation and only to a small extent the variability among replicate tanks^58^.

During the OAW & N experiment (summer 2014), the mean temperature (±SD) under ambient conditions was 19.3 ± 2.4 °C and under warmed condition 23.6 ± 2.7 °C. The mean difference between the warmed and ambient conditions was 4.4 ± 0.6 °C. Mean pH (±SD) under ambient conditions was 7.95 ± 0.13 and under acidified conditions 7.63 ± 0.13. The mean difference in pH between the ambient and the acidified treatment was 0.32 ± 0.14. The mean salinity (±SD) under ambient conditions was 16.5 ± 1.5 (Fig. S3). During the local upwelling event, mean temperature (±SD) was 16.4 ± 0.3 °C and the mean O_2_ concentration (±SD) was 2.71 ± 0.37 mg O_2_ L^−1^ (Fig. S4).

## Supplementary information

Suppl. Table 1

## Data Availability

All relevant raw data have been deposited in the PANGAEA repository (https://doi.org/pangaea.de/10.1594/PANGAEA.900089).
