# Supplementary material for: Sensitivities to global change drivers may correlate positively or negatively in a foundational marine macroalga
Source: Sci Rep. 2019 Oct 10;9:14653. doi: 10.1038/s41598-019-51099-8 (PMC6787226; doi:10.1038/s41598-019-51099-8)
Supplement: Supplementary file 1 — Suppl. Table 1 [file 41598_2019_51099_MOESM1_ESM.docx]

*Supplementary material to the following paper:*

**Sensitivities to global change drivers may correlate positively or negatively in a foundational marine macroalga.**

Balsam Al-Janabi^1^, Martin Wahl^2*^, Ulf Karsten^3^, Angelika Graiff^4^, Inken Kruse^5^

^1^ GEOMAR Helmholtz Centre for Ocean Research Kiel, Department of Marine Ecology, Duesternbrooker Weg 20, D-24105 Kiel, Germany, baljanabi@gmx.de

^2^ GEOMAR Helmholtz Centre for Ocean Research Kiel, Department of Marine Ecology, Duesternbrooker Weg 20, D-24105 Kiel, Germany, mwahl@geomar.de

^3^ University of Rostock, Institute of Biological Sciences, Applied Ecology and Phycology, Albert-Einstein-Strasse 3, D-18059 Rostock, Germany, ulf.karsten@uni-rostock.de

^4^ University of Rostock, Institute of Biological Sciences, Applied Ecology and Phycology, Albert-Einstein-Strasse 3, D-18059 Rostock, Germany, angelika.graiff@uni-rostock.de

^5^ GEOMAR Helmholtz Centre for Ocean Research Kiel, Department of Marine Ecology, Duesternbrooker Weg 20, D-24105 Kiel, Germany, inkenkruse@web.de

* Corresponding author phone +49 4316004500, Fax: +49 4316001671, E-mail: mwahl@geomar.de

**Supplementary Table 1** Nutrient concentrations in µmol L^-1^ in the ambient (mean of the last 7 years of the respective months) and future nutrient conditions (doubled amounts of the ambient nutrient concentrations) for NO_2_, NO_3_ PO_4_.

|  |  | July | |  | August | |  | September | |
| --- | --- | --- | --- | --- | --- | --- | --- | --- | --- |
|  |  | Ambient | Future |  | Ambient | Future |  | Ambient | Future |
| NO_2_ |  | 0.53 | 1.05 |  | 0.77 | 1.54 |  | 1.27 | 2.54 |
| NO_3_ |  | 0.18 | 0.36 |  | 0.20 | 0.40 |  | 0.22 | 0.44 |
| PO_4_ |  | 0.46 | 0.93 |  | 0.59 | 1.19 |  | 1.06 | 2.11 |
